# Gestational Age Variation in Human Placental Drug Transporters

**DOI:** 10.3389/fphar.2022.837694

**Published:** 2022-04-06

**Authors:** Laura Goetzl, Nune Darbinian, Nana Merabova, Lindsay C. Devane, Sammanda Ramamoorthy

**Affiliations:** ^1^ Department of Obstetrics, Gynecology and Reproductive Sciences, McGovern Medical School at The University of Texas Health Science Center at Houston (UTHealth), Houston, TX, United States; ^2^ Center for Neural Repair and Rehabilitation, Shriners Hospitals Pediatric Research Center, Lewis Katz School of Medicine at Temple University, Philadelphia, PA, United States; ^3^ Department of Family Medicine, Medical College of Wisconsin-Prevea Health, Green Bay, WI, United States; ^4^ Department of Psychiatry, Medical University of South Carolina, Charleston, SC, United States; ^5^ Department of Pharmacology and Toxicology, Virginia Commonwealth University, Richmond, VA, United States

**Keywords:** placental transporters, serotonin transporter (5HTT), P-glycoprotein (ABCB1 protein), multidrug resistance protein 3 (MDR3), breast cancer resistance protein (BCRP or ABCG2), norepinephrine transporter, placental drug transport

## Abstract

Patient and providers’ fear of fetal exposure to medications may lead to discontinuation of treatment, disease relapse, and maternal morbidity. Placental drug transporters play a critical role in fetal exposure through active transport but the majority of data are limited to the 3rd trimester, when the majority of organogenesis has already occurred. Our objective was to define gestational age (GA) dependent changes in protein activity, expression and modifications of five major placental drug transporters: SERT, P-gp, NET, BCRP and MRP3. Apical brush border membrane fractions were prepared from fresh 1st, 2nd and 3rd trimester human placentas collected following elective pregnancy termination or planned cesarean delivery. A structured maternal questionnaire was used to identify maternal drug use and exclude exposed subjects. Changes in placental transporter activity and expression relative to housekeeping proteins were quantified. There was evidence for strong developmental regulation of SERT, NET, P-gp, BCRP and MRP3. P-gp and BCRP decreased with gestation (r = −0.72, *p* < 0.001 and r = −0.77, *p* < 0.001, respectively). Total SERT increased with gestation but this increase was due to a decrease in SERT cleavage products across trimesters. Uncleaved SERT increased with GA (r = 0.89, *p* < 0.001) while cleaved SERT decreased with GA (r = −0.94, *p* < 0.001). Apical membrane NET overall did not appear to be developmentally regulated (r = −0.08, *p* = 0.53). Two forms of MRP3 were identified; the 50 kD form did not change across GA; the 160 kD form was steady in the 1st and 2nd trimester and increased in the 3rd trimester (r = 0.24, *p* = 0.02). The 50 kD form was expressed at higher levels. The observed patterns of SERT, NET P-gp, BCRP and MRP3 expression and activity may be associated with transporter activity or decreased placental permeability in the 1st trimester to transporter specific substrates including commonly used psychoactive medications such as anti-depressants, anti-psychotics, and amphetamines, while transport of nutrients and serotonin is important in the 1st trimester. Overall these observations are consistent with a strong protective effect during organogenesis. 3rd trimester estimates of fetal exposure obtained from cord blood likely significantly overestimate early fetal exposure to these medications at any fixed maternal dose.

## Introduction

Psychoactive medications are commonly used throughout pregnancy. Approximately 10–25% of pregnant women have a major psychiatric disorder either major depression, or bipolar disorder, schizophrenia, or an anxiety disorder (Gavin et al., 2005; Fawcett et al., 2019). Prevalence of use in pregnancy has been reported to be 5.5% for selective serotonin reuptake inhibitors (SSRIs) and 2.0% for benzodiazepines (Molenaar et al., 2020; Qato and Gandhi, 2021). In addition, the use of amphetamines for hyperactivity disorders, weight loss, and to enhance cognitive performance is widespread, especially in young women at peak fertility. The prevalence of prescription amphetamine (e.g., Adderall) use in college age adults is 2.1–5.8% (McCabe et al., 2005, therefore amphetamines are one of the most common drug exposures in early pregnancy. A major concern of drug administration during pregnancy is unintended effects on the fetus (Cragan et al., 2006; Sharma et al., 2008).

Patient and providers fear of fetal exposure to medication may lead to discontinuation of treatment, disease relapse, and significant maternal morbidity. Treatment of mental illness results in 1st trimester exposure to an array of drugs during fetal organ development, and fetal brain development continues through the 2nd and 3rd trimesters. The consequences of fetal drug exposure may be inconsequential, disastrous, or, in most cases, unknown. Ultimately, it is difficult to evaluate the dose-effect relationship of drugs on fetal development without an accurate estimate of dose across all three trimesters. In the past, we have reviewed how placental drug transporters play a critical role in fetal exposure though active transport (Wang et al., 2007; DeVane et al., 2011). Transporters can be influx transporters (maternal to fetal) or efflux transporter (fetal to maternal). Key placental drug transporters and their clinically relevant substrates are summarized (Table 1; International Transporter Consortium, 2010).

**TABLE 1:** Placental transporters and their clinical relevance in pregnancy.

Transporter	Placental location and directionality	Substrates commonly used in young women	Inhibitors commonly used in young women
P-glycoprotein	Apical Membrane Efflux	Anti-depressants: citalopram, amitriptyline, paroxetine, venlafaxine	Calcium Channel Blockers (Verapamil)
		Anti-psychotics: aripiprazole (aripiprazole, quetiapine, olanzapine, paliperidone, chlorpromazine, Risperidone, Clozapine)	Erythromycin
			Ceftriaxone
		Opioids: Methadone, Morphine	Progesterone
		Other: Glucocorticoids, Glyburide, Phenytoin, protease Inhibitors, Ondansetron, Cimetidine/ranitidine	Ketoconazole
			Protease Inhibitors
			Beta Blockers
			Hydrocortisone
Multidrug Resistance Protein 3	Apical Membrane Efflux	Bile Salts	Indomethacin
		Morphine	
		Acetaminophen	
		Fexofenadine	
Breast Cancer Resistance Protein	Apical Membrane Efflux	Antipsychotics: Risperidone, paliperidone	Reserpine
		Erythromycin, Bile Acids	Protease Inhibitors
		Nitrofurantoin, Bupropion	Zidovudine
		Cimetidine	Acetaminophen
		Fluoroquinolones	
Norepinephrine Transporter	Apical Membrane Influx	Catecholamine	Norepinephrine **>** Serotonin Reuptake Inhibitors
	Basal Membrane Influx	Amphetamines	
Serotonin Transporter	Apical Membrane Influx	Serotonin, Amphetamine, Methylphenidate, MDMA, Fenfluramine, Norfenfluramine	Serotonin Reuptake Inhibitors
			Tricyclic antidepressants
			Cocaine

Most data about placental transporters have been derived from placental tissues collected in the 3rd trimester at birth, as it is difficult to collect healthy placental tissue in the 1st and 2nd trimester. Ironically, these trimesters are when most organogenesis occurs. In addition, potential changes in the proportion of cellular membranes to connective tissue across trimesters complicate direct comparison of protein levels across trimesters. The aim of this study was to provide a comprehensive assessment of protein levels and activity across gestation in comprehensive group of proteins that are known to be involved in placental transport of those psychoactive medications that are most common in pregnancy: Serotonin Transporter (SERT), P-glycoprotein (P-gp), Norepinephrine Transporter (NET), Breast Cancer Resistance Protein (BCRP) and Multidrug Resistance Protein 3 (MRP3). Substantial innovations in the current study include 1) a structured patient interview to exclude patients with known exposure to psychoactive medication exposure 2) assessment of protein levels from apical membrane fractions instead of whole placental extracts 3) demonstrated retention of active protein function 4) assessment of all transporters in the same large set of samples, minimizing unnecessary variability and 5) identification of developmental stage-specific forms of certain drug transporters.

## Methods

### Clinical Recruitment

1st and 2nd trimester placental tissue was collected from women undergoing elective pregnancy termination under an IRB approved protocol. 3rd trimester placental tissue was collected following scheduled pre-labor cesarean delivery. A face to face history was conducted by a trained study coordinator and an extensive medication and substance exposure history was obtained. Subjects of this study denied any medical conditions, exposure to medications, ethanol, tobacco or street drugs. Subjects with known fetal anomalies, multiple gestations, incomplete miscarriage or fetal anomalies were excluded. The 1st trimester samples were collected between 8 and 13 weeks, 2nd trimester samples between 14 and 20 weeks and 3rd trimester samples were largely >37 weeks’ gestation.

### Tissue Handling and Preparation of Brush Border Membrane Vesicles

Fresh tissue was immediately rinsed in cold phosphate-buffered saline (PBS) to remove any blood or decidua. Villous tissue was placed on ice and transported immediately from the clinical setting to the laboratory. Time from collection to processing was consistently <1 h. Apical brush border membrane fractions were prepared according to our published protocols by Ramamorthy with minor modifications (Prasad et al., 1996). Briefly, tissue was cut into small pieces, washed and rinsed with wash buffer three times (10 mM HEPES, 300 mM mannitol, adjust pH with Tris to 7.0, protease inhibitors cocktail 1:100 dilution). For 3rd trimester placenta, filtrates were removed using cheesecloth. Rinsed pieces were macerated using razor blade to expose the synctiotrophoblast brush-border membrane and to remove other parts of the tissue, then collected and placed in an ice-cold beaker. The tissue was placed in cold wash buffer and agitated for 30 min at 4°C. The filtrate was centrifuged at 7,000 rpm for 15 min. The pellet was discarded, and the supernatants were centrifuged at 25,000 rpm at 4°C for 30 min. The pellet was washed with cold buffer and homogenized in ice, then samples were placed in wash buffer in a small pre-chilled beaker with stir bar with MgCl2 (10 mM final concentration) for 1 min and incubate on ice for 10 min. The suspension was centrifuged at 4,300 rpm for 15 min at 4°C. The pellet was discarded and the supernatants were centrifuged again at 25,000 rpm for 30 min at 4°C. The pellet was homogenized again using 25-G syringe to make suspension. Samples were transferred into new centrifuge tube with a buffer, centrifuged for 30 min at 25,000 rpm and the pellet was resuspended in preloading buffer. Protein concentration was determined using Bradford assay. Aliquots were stored in liquid nitrogen for further protein analysis by western-blot assay and transporter activity assay. Right side out (ROV) and inside out (IOV) vesicles were created depending on whether or not an efflux or influx transporter was assessed.

### Alkaline Phosphatase Activity

The purity of brush border membrane preparations was assessed by ALP activity using a colorimetric (spectrophotometric) enzymatic ALP Assay Kit (Abcam) in duplicates according to the manufacturer’s instruction. ALP catalyzes the hydrolysis of phosphate esters in alkaline buffer and produces an organic radical and inorganic phosphate. Optical density at 405 nm was measured using ELx808 microplate reader (Bio-Tec) and Gen5 software. P-nitrophenyl phosphate (pNPP) was used as a phosphatase substrate which turns yellow (λ_max_ = 405 nm) when dephosphorylated by ALP. The pNPP standards were prepared and incubated with ALP enzyme for 60 min to generate the standard curve (0–20 nmol/reaction). Samples were incubated with pNPP for 60 min before adding stop solution. Results were determined by comparing the value of the sample to standard whose value is known by using the standard curve to determine the concentration of the metabolite in each sample**.** Average enzyme activity was normalized to total protein: ALP activity (U/ml) = pNP generated by sample (in μmol)/volume of sample in reaction (ml)/reaction time (minutes).

### SERT Activity

Using ROV, we conducted SERT mediated serotonin (5-HT) transport using radiolabeled 5-Hydroxy-[^3^H] tryptamine creatinine sulfate ([^3^H]5-HT). Nonspecific [^3^H]5-HT uptake was defined as the accumulation in the presence of 10 nM fluoxetine and was subtracted from the total counts and SERT-specific 5-HT transport was confirmed.

### NET Functional Activity

Using ROV,NET-mediated norepinephrine (NE) uptake and the level of NET protein expression were determined using radiolabeled NE and NET specific antibody.

### P-gp Activity

Using IOV, P-gp specific [^3^H]-paclitaxel transport was assessed in the presence and absence of ATP generating media and the P-gp inhibitor, verapamil.

### Protein Quantification

Quantitative western blotting was performed as previously described (Darbinian et al., 2014). In brief, protein extracts were prepared from apical membrane fractions. Fifty micrograms of protein was diluted with Laemmli SDS-sample reducing buffer, (6X), heated at 95°C for 10 min and separated by gradient SDS-PAGE gel electrophoresis in 1X Tris-Glycine-Sodium Dodecyl Sulfate buffer and transferred to nitrocellulose membrane (Bio-Rad) and blotting papers (Grade GB003) for 2 h at 4°C. The blots were subsequently washed three times and proteins were detected using specific primary antibodies. Blots were then incubated with IRDye® 800CW Goat Anti-Rabbit and IRDye® 680RD Goat Anti-Mouse Li-COR dyes and visualized/quantitated with an Odyssey® CLx Imaging System (LI-COR, Inc., Lincoln, NE) using Odyssey software (LI-COR Biosciences, Lincoln, NE, United States). Plate-based assay techniques (Enzyme or Enzyme Linked Immunosorbent Assays) were used to detect and quantify proteins in ROV or IOV. Assays were performed per the manufacturer’s instructions. The following kits were used: Human SERT (NeoBiolab, Cambridge, MA), Human NET (MyBioSource, San Diego, CA), and Human P-gp (Antibody Research, St Charles, MO).

### Antibodies

The following antibodies were used. Anti-SERT rabbit polyclonal antibody/AB10514P (EMD Millipore, Billerica, MA); anti-P-gp mouse monoclonal antibody Clone 2F7 (OriGene, Rockville, MD); anti-MRP3 rabbit anti-human antibody LS-C177398 (Lifespan Biosciences Inc, Seattle, WA); anti-NET rabbit polyclonal antibody LS-C101935 (Lifespan Biosciences Inc, Seattle, WA) and anti-BCRP mouse monoclonal antibody BXP-21 (Abcam, Cambridge, MA). Loading control antibodies included: Anti-GAPDH (6C5, sc-32233, Santa Cruz Biotechnologies, Santa Cruz, CA); mouse monoclonal Grb2 (BD Biosciences, San Jose, CA), and anti-α-Tubulin clone B512 (Sigma-Aldrich Co, St. Louis, MO). Primary antibodies were diluted according to the manufacture’s suggestion (1:1,000). IRDye® 800CW Goat Anti-Rabbit and IRDye® 680RD Goat Anti-Mouse Li-COR dyes (LI-COR, Inc., Lincoln, NE) were diluted at 1:20,000. RFU readings were normalized to control housekeeping proteins (Grb2, Tubulin, or GAPDH) in qWestern-blot assays.

### SERT Sequencing

For the sequencing of N-terminal residues, 50 μg of placenta vesicles isolated from 1st trimester control samples were used for SDS-PAAG gel electrophoresis, then proteins were transferred onto PVDF membrane and stained with Coomassie Blue solution. Membranes were washed with water to remove the glycine. Bands with size corresponding to 34–50 kDa molecular weight were cut and the Edman degradation experiment was performed to get the N-terminal sequence of truncated SERT protein versions (CD Biosciences Inc., Shirley, NY, www.creative-biolabs.com). An ABI Procise 494 sequencer was used. Sequences were compared with two 5HT transporter isoforms downloaded from Unipro. The sequence alignment was performed using ClustalW2 on two isoforms of SERT and potential N-terminal fragments (http://www.uniprot.org/uniprot/P31645#P31645, gene names SLC6A4; Synonyms: HTT, SERT; Organism *Homo sapiens* (Human), Taxonomic identifier9606 [NCBI]). The 1st four amino acids on the N-terminal region of SERT were detected in two potential N-terminal fragments. In combination with the western blot results, the presence of truncated SERT protein in 1st trimester placental vesicles was confirmed.

### Statistical Analysis

Statistical analysis was performed on IBM SPSS Statistics for Windows, Version 22.0 (Armonk, NY: IBM Corp). Means were compared using ANOVA with post-hoc LSD testing and medians were compared using the sign log rank test. Correlations were performed using the Spearman test. A *p*-value of <0.05 was considered significant.

## Results

A total of 96 samples were collected: 33 in the 1st trimester, 32 in the 2nd trimester and 21 in the 3rd trimester.

### ALP

**FIGURE 1:**
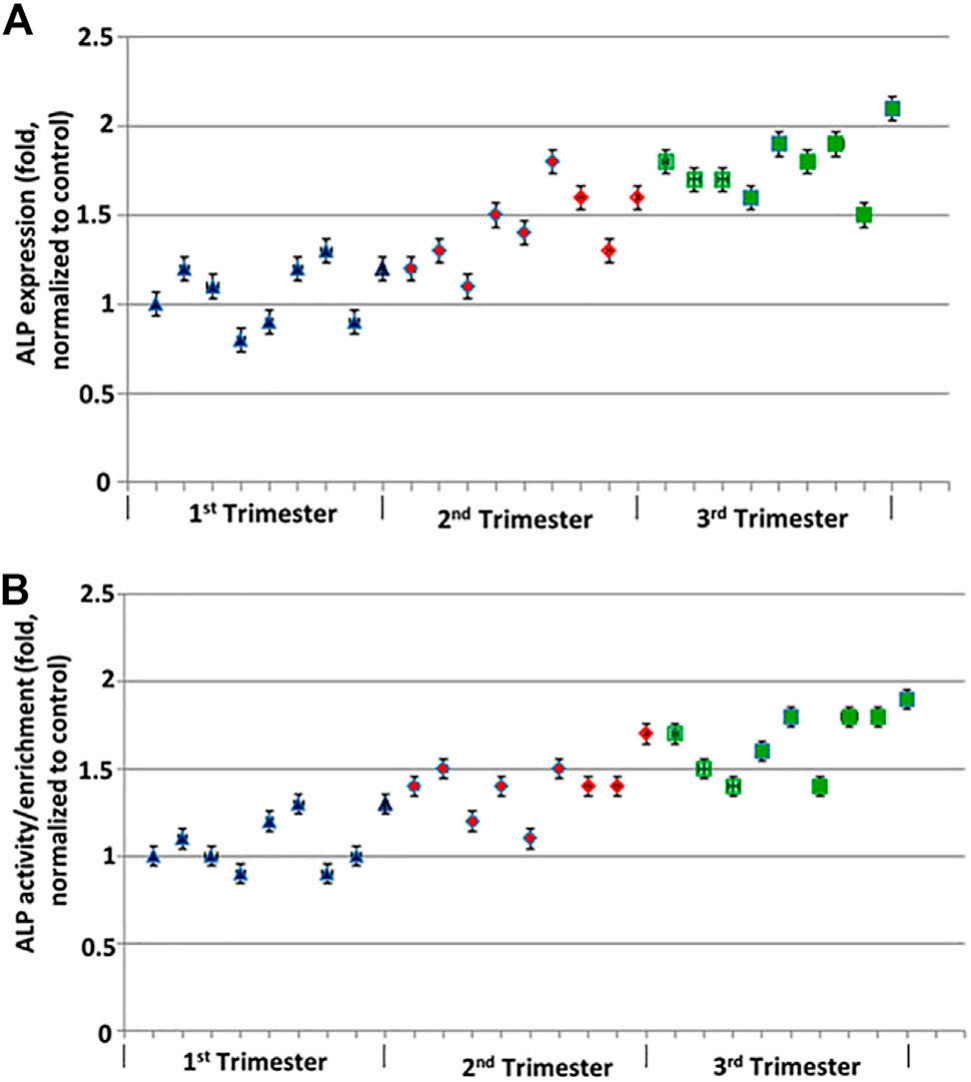
ALP protein expression across GA normalized to Grb2. **(A)** Western blot analysis was performed for the expression for ALP. ALP protein levels were quantified using LICOR platform. **(B)** ALP activity across GA by ABCAM colorimetric assay compared to pNPP. Error bars represent standard deviation of triplicate readings.

Membrane expression of ALP protein was demonstrated by western blotting and suggested increased ALP expression with increasing GA (Figure 1A). Highest enrichment of ALP activity was evident in brush border membrane preparations (Figure 1B). Both protein levels and activity of ALP increase with increasing GA. Average enzyme activity at A405 (normalized to total protein) was 0.03 U/ml. Average ALP activity in placental homogenates was 4.1 U/mg/min, while average ALP activity in purified vesicle fractions was 176.1 U/mg/min, suggestion an average enrichment of 42.6-fold.

### SERT

**FIGURE 2:**
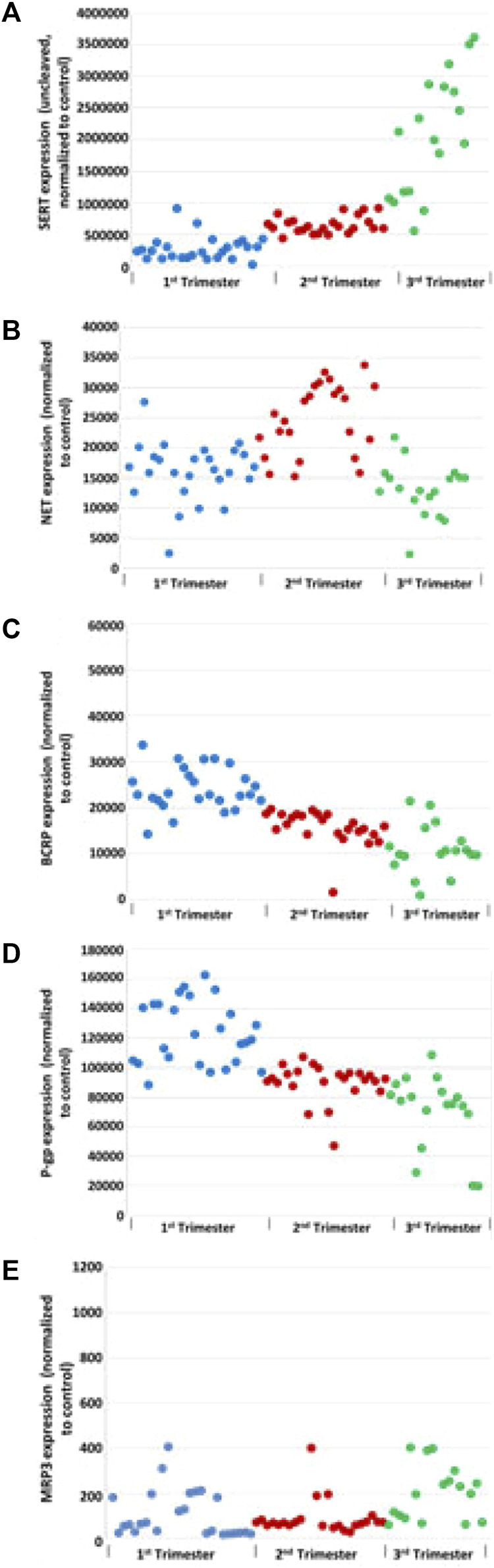
Effect of GA on transporters levels: total SERT **(A)**, NET **(B)**, BCRP **(C)**, P-gP **(D)**, and MRP3 **(E)** in human placental membrane vesicles by Western blot analysis. Grb2 served as a loading control.

**FIGURE 3:**
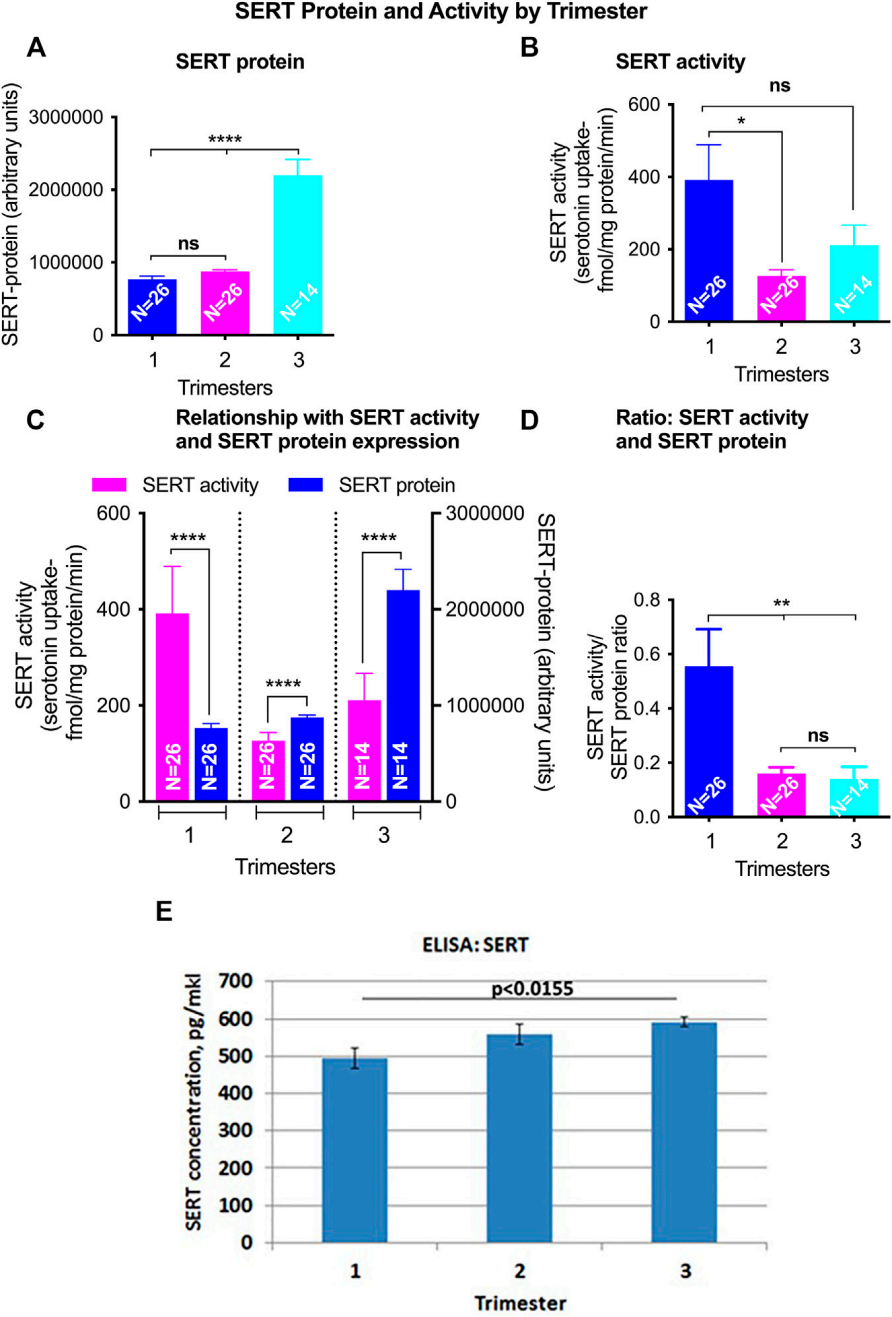
GA differences in total SERT levels and activity in ROV placental vesicles. Total SERT quantified by Western blot in matched samples used for activity assays **(A)**. SERT mediated serotonin (5-HT) transport was quatified using radiolabelled 5-Hydroxy-[^3^H] tryptamine creatinine sulfate ([^3^H]5-HT). Nonspecific [^3^H]5-HT uptake was defined as the accumulation in the presence of 10 nM fluoxetine and was subtracted from the total counts **(B)**. The relationship of SERT mediated 5-HT transport and SERT protein levels from the same membrane vesicles across gestations was analyzed **(C,D)**. Levels of SERT transporter in ROV were also assayed by ELISA [**(E)**; pg/mkl of vesicles]. Symbols represent the following levels of statistical significance: *p < 0.05, **p < 0.01, ***p < 0.001.

Total SERT protein levels increased across GA (r = 0.69, *p* < 0.001, Figure 2A) by quantitative western blotting. There was a moderate rise in high molecular weight SERT (70 kDa) levels in the 2nd trimester but a sharp increase in the 3rd trimester. Median normalized levels ± interquartile range (IQR) were 706,747 ± 133,626 (RFU) in the 1st trimester, 851,933 ± 133,292 (RFU) in the 2nd trimester and 2,035,086 ± 1,125,016 (RFU) in the 3rd trimester (*p* < 0.001). Significant changes were found in ROV SERT levels by ELISA between the 1st and 3rd trimester (*p* < 0.0155) (Figure 3).

#### SERT Isoforms

**FIGURE 4:**
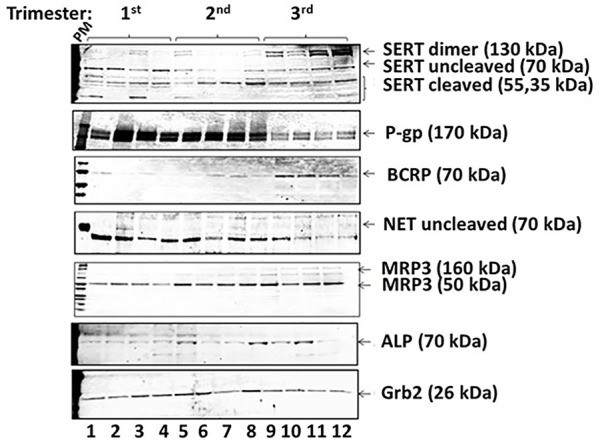
Exemplar of Western blot results from placenta vesicles from 1st, 2nd and 3rd trimesters (*n* = 4 per trimester) for SERT and its isoforms, P-gp, BCRP, NET, MRP3, and ALP. Grb2 served as a loading control.

**FIGURE 5:**
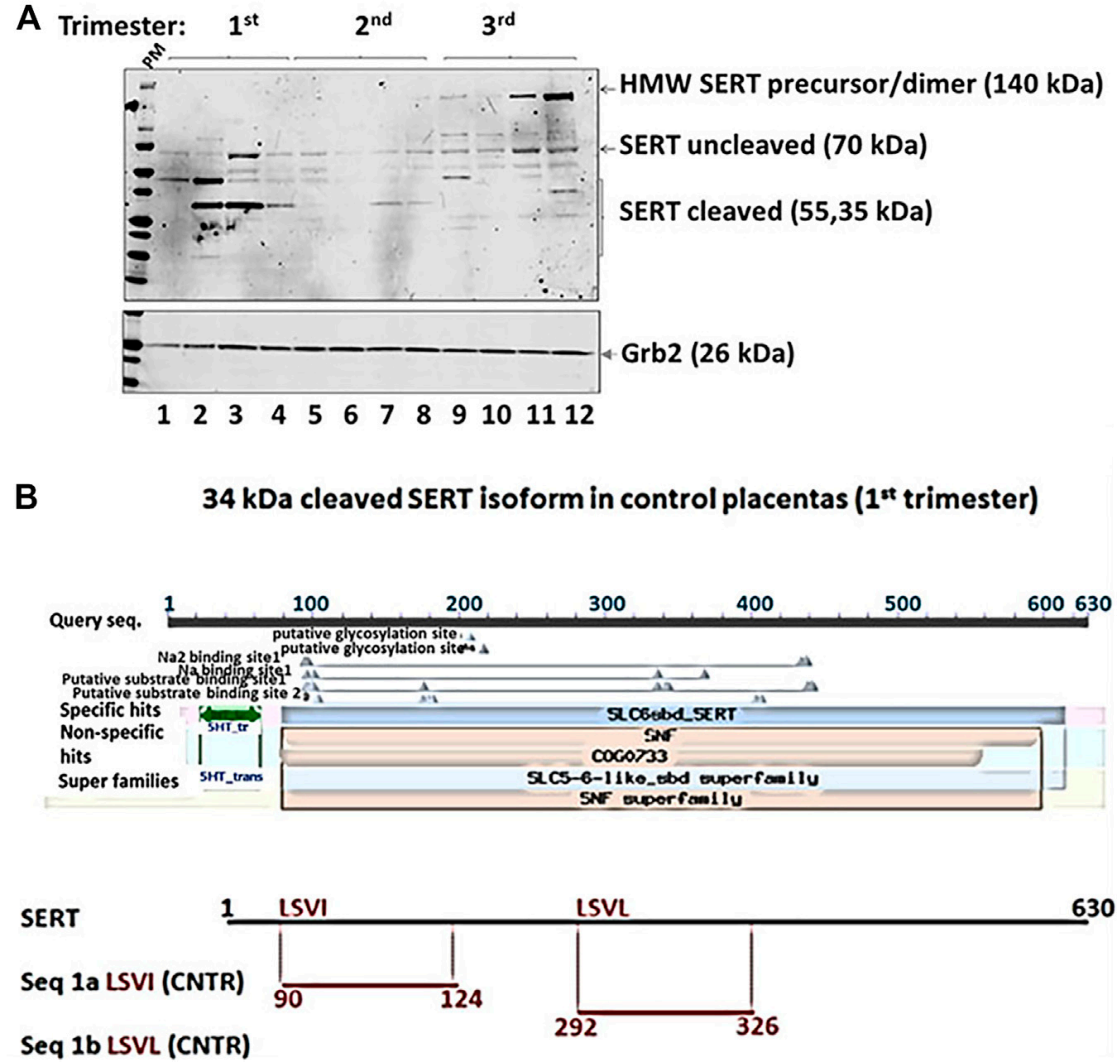
Edman degradation was performed to obtain the N-terminal sequence of truncated SERT protein versions. Two potential N-terminal SERT fragments were present in the sample **(A)**. The sequences were compared with two 5HT transporter isoforms downloaded from Unipro **(B)**. The sequence alignment was performed using ClustalW2 on two isoforms of SERT and potential N-terminal fragments on SLC6A4 (Synonyms: HTT, SERT; Organism *Homo sapiens* (Human), Taxonomic identifier9606 [NCBI]).

We identified a previously undescribed slightly smaller isoform of the 70 kDa form of SERT that was prominent in 1st trimester samples (Figures 4, 5). There was a strong correlation between GA and subsets of cleaved and uncleaved SERT. Uncleaved SERT increases with GA (r = 0.89, *p* < 0.001) while cleaved SERT decreases with GA (r = −0.94, *p* < 0.001). Via sequencing, we found that the truncated version starts from amino acid LSVI 90 (out of 630) of human SERT isoform (Figure 5B). Antibody against the central region of SERT immunoreacted with a band that starts from a 292, and antibody specific for N-terminal region immunoreacted with a form that starts from a 90.

### NET

NET levels (70 kDa) did not appear to be developmentally regulated (Figure 2B). There was no linear correlation between GA and NET levels (r = −0.08, *p* = 0.53). Median levels were 16,616 ± 4,757 in the 1st trimester, 25,060 ± 11,030 in the 2nd trimester and 12,849 ± 6,030 in the 3rd trimester. The increased levels seen in the 2nd trimester were statistically significant (*p* < 0.001 compared to 1st trimester, *p* < 0.001 compared to 3rd trimester). Less NET was expressed at 3rd trimester. NET isoform patterns did not change across gestation (Figure 4).

**FIGURE 6:**
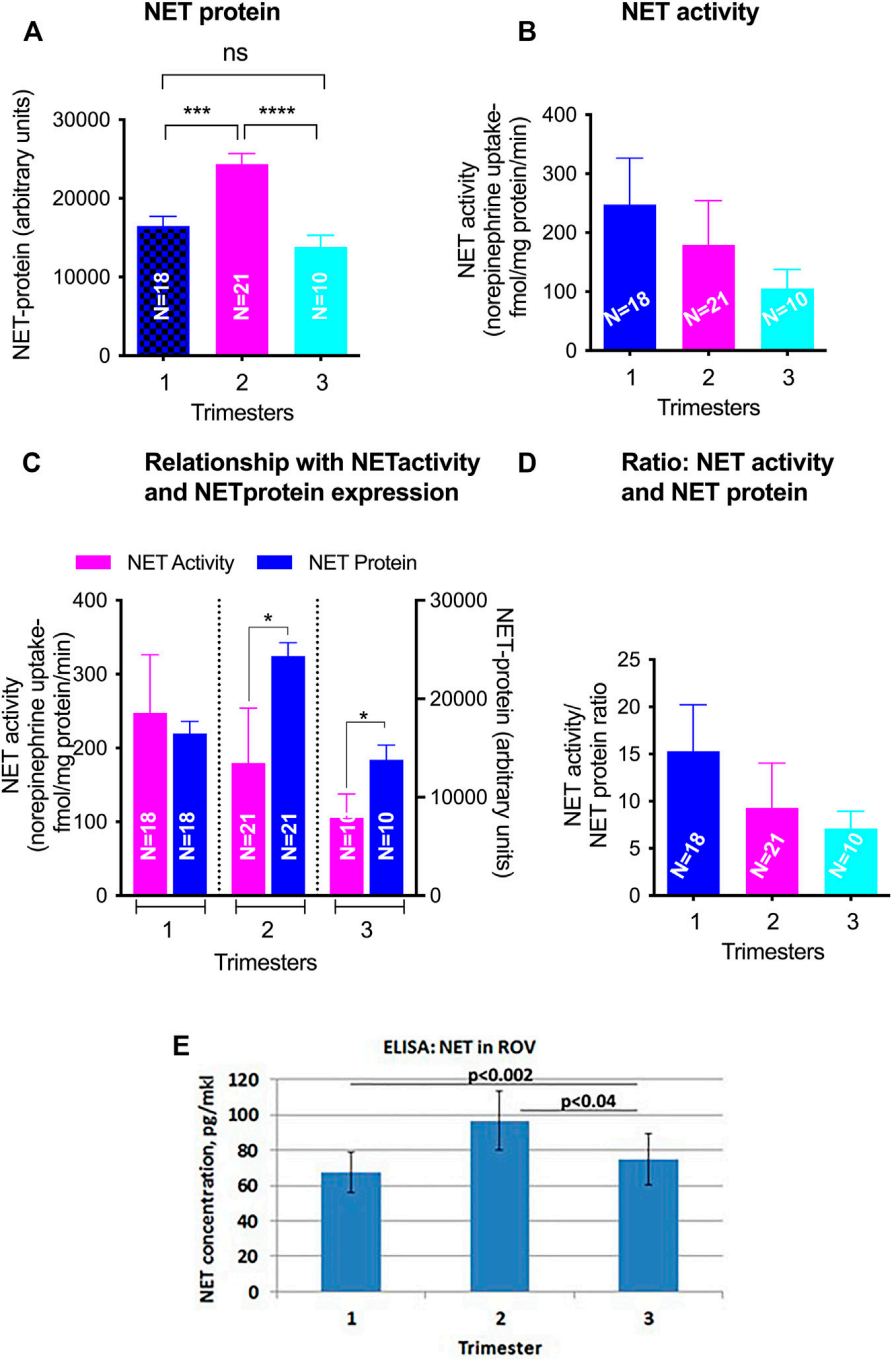
GA differences in total NET levels and activity in ROV placental vesicles. Total NET quantified by Western blot in matched samples used for activity assays **(A)**. NET-mediated norepinephrine uptake and the level of NET protein expression was determined using radiolabelled NE and NET specific antibody **(B)**. The relationship of SERT mediated 5-HT transport and SERT protein levels from the same membrane vesicles across gestations was analyzed **(C,D)**. Levels of SERT transporter in ROV were also assayed by ELISA [**(E)**; pg/mkl of vesicles]. Symbols represent the following levels of statistical significance: *p < 0.05, ***p < 0.001, ****p < 0.001.

Based on ELISA data, statistically significant changes were found in NET levels in ROV in groups between 2nd and 3rd trimester (*p* < 0.04), and between 1st and 3rd trimester (*p* < 0.002) (Figure 6E).

### BCRP

BCRP levels decreased linearly across gestation (Figure 2C, r = −0.77, *p* < 0.001). Median normalized levels were 231,050 ± 8,589 RFU in the 1st trimester, 16,738 ± 4,253 in the 2nd trimester and 10,355 ± 5,057 in the 3rd trimester (*p* < 0.001). Exemplars of western blots for BCRP are shown (Figure 4). BCRP appears as a single band at 70 kD as expected (Volk and Schneider, 2003; Mao, 2008; Vähäkangas and Myllynen, 2009). No changes in BCRP isoforms were identified across gestation.

### P-gp

**FIGURE 7:**
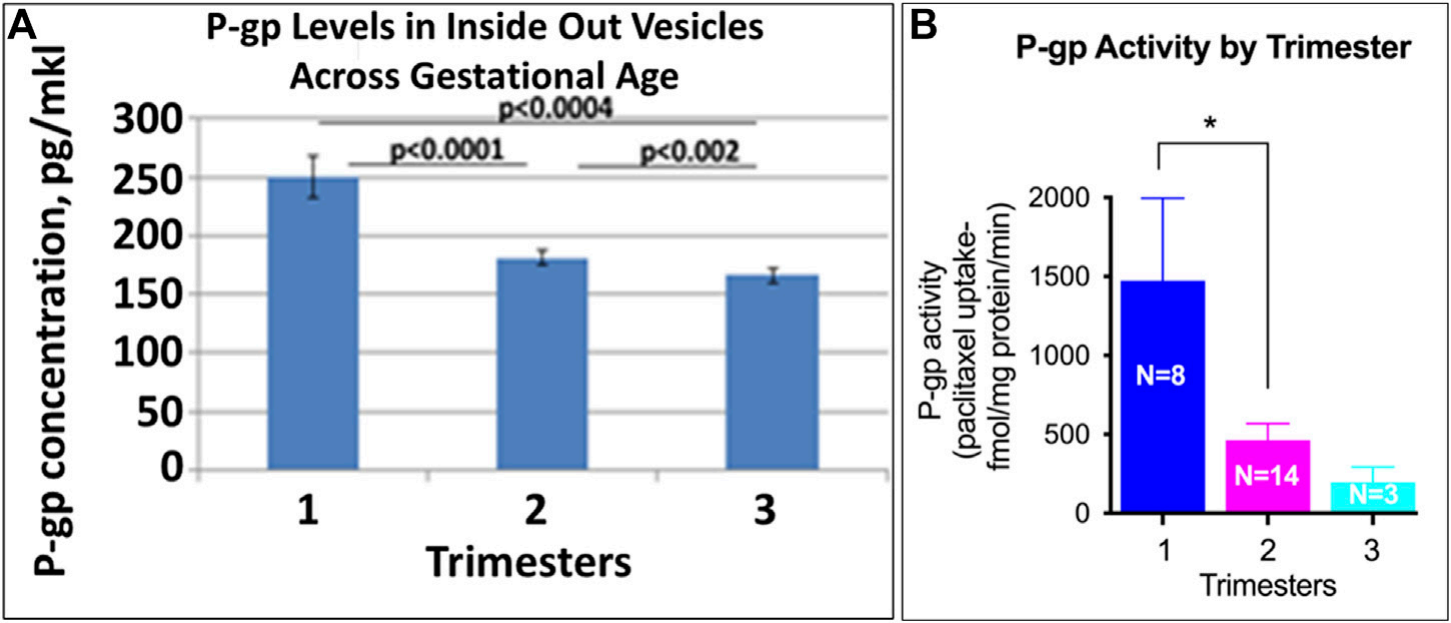
Placental P-gp activity across GA. Levels of P-gp in IOV were assayed by ELISA (**A**; pg/mkl of vesicles). P-gp specific [^3^H]-paclitaxel transport was validated in the presence and absence of ATP generating media and the ability of P-gp inhibitor, verapamil to block [^3^H]-paclitaxel transport **(B)**. Symbols represent the following levels of statistical significance: *p < 0.05.

P-gP appears as a double band at ∼170 kD (Figure 4). P-gP levels decreased linearly across gestation (Figure 2D, r = −0.72, *p* < 0.001). Median normalized levels were 116,849 ± 41,488 RFU in the 1st trimester, 92,645 ± 11,745 in the 2nd trimester and 76,427 ± 22,136 in the 3rd trimester (*p* < 0.001). Of interest, variability was greatest in the 1st trimester. No degradation or smaller bands were observed and no changes in P-gP isoforms were identified. Based on ELISA data, statistically significant changes were found in P-gP levels in IOV in all groups between 1st and 2nd trimester (*p* < 0.0001), between 2nd and 3rd trimester (*p* < 0.002), and between 1st and 3rd trimester (*p* < 0.0004) (Figure 7A).

### Multidrug Resistance Protein 3 (MRP3, ABCC3)

Two isoforms of MRP3 were identified, a high molecular weight 160 kDa form and a low molecular weight 50 kDa form (Figure 4). Total MRP3 levels were variable did not appear to be developmentally regulated (r = −0.02, *p* = 0.88, Figure 2E). When the two isoforms were examined separately, median levels of the 50 kDa form were 5,513 ± 13,262 in the 1st trimester, 3,563 ± 6,138 in the 2nd trimester and 4,442 ± 19,804 in the 3rd trimester (*p* = 0.42). In contrast levels of the 160 kDa isoform increased in the 3rd trimester. Median levels were 414.7 ± 1,363.9 in the 1st trimester, 174.8 ± 621.2 in the 2nd trimester and 872.0 ± 3,335.9 in the 3rd trimester (*p* = 0.02; 3rd trimester vs. 1st trimester, *p* = 0.02; 3rd trimester vs. 2nd trimester, *p* = 0.01; 1st vs. 2nd trimester *p* = 0.88). While developmental differences in the 160 kDa isoform expression may affect drug transport, the 50 kD isoform appears to be predominant and is expressed at ∼5 times higher levels; the impact of the effect will be influenced by the relative *in vivo* transport potential of each isoform.

## Discussion

Our investigations provide evidence that developmental regulation of placental drug transporters is extremely complex. In broad strokes, the efflux transporters P-gp and BCRP appear to be maximally expressed in the 1st trimester. In combination, this scenario would result in maximal fetal protection in the earliest gestational periods at the time of organogenesis. Fetal exposure to substrates of these transporters may increase over gestation at the time of 3rd trimester fetal brain development and synaptogenesis. The potential effects of this exposure, if any, are not well understood. Increasing placental transport may also be balanced by an increasing ability by the fetus to metabolize drugs and other xenobiotics; thereby lessening their impact (Prouillac and Lecoeur, 2010). In contrast, our data suggest that the activity of the influx transporter SERT appears to be highest in the 1st trimester, suggesting fetal vulnerability early in development and decreased vulnerability during 3^rd^ trimester brain development.

### SERT

SERT is responsible for the transport of serotonin and selective serotonin reuptake inhibitors (SSRIs) inhibit SERT function. In addition, SERT is a major transporter of amphetamines and related compounds that competitively inhibit serotonin transport (Ramamoorthy JD. et al., 1995). SERT has long been known to be expressed in the apical membranes in 3rd trimester placental tissue (Balkovetz et al., 1989; Ramamoorthy et al., 1993a) and an *in vitro* placental cell lines (Cool et al., 1991). Recently, Anoshchenko also quantified GA-dependent protein levels of SERT in cellular membrane fractions from the placenta (Anoshchenko et al., 2020). They performed quantitative targeted proteomics and reported a non-significant decrease in SERT with increasing gestation, however their investigation was in a smaller sample size (≈15 per trimester), was unable to assess various isoforms, and did not asses transporter activity. In contrast, our data indicate that SERT expression is low in the 1st trimester, but increases with GA, especially in the 3rd trimester of pregnancy; only the shorter isoform decreases with GA. In theory, low 1st trimester levels of the SERT precursor with various phosphorylation would at least partially protect the fetus from the effects of exposure from competitive substrates such as amphetamines (Ramamoorthy S. et al., 1995). SERT activity assays demonstrated higher activity in early trimesters, suggesting that N-terminal cleaved SERT is the active form. These data are relevant to young women who experience unintended pregnancy while using amphetamines or related substances for treatment of ADHD, weight loss, or to enhance school performance. In contrast, our data highlight the potential for decreasing fetal vulnerability to amphetamines and other substrates in the 3rd trimester of pregnancy.

Serotonin transport in pregnancy is a delicate balance; patients undergoing assisted reproduction who carry the LL genotype for 5-HTTLPR in the serotonin transporter promoter, which results in increased levels of SERT, had lower clinical pregnancy rates and increased rates of biochemical pregnancy loss (Palomares et al., 2013). Prenatal exposure to bisphenol A increases hippocampal SERT in a murine model (Matsuda et al., 2013). In contrast, cannabinoids exposure has been associated with decreased SERT transporter activity *in vitro* (Kenney et al., 1999). Therefore, self-medication with cannabanoids in the 1st trimester for pregnancy-associated nausea may lower fetal delivery of SERT with unknown consequences. Similarly, co-exposure to SSRIs may impact fetal serotonin levels across GA.

Finally, we report novel findings regarding SERT isoforms across GA. We have demonstrated a marked increase in SERT cleavage products in the 1st trimester. This suggests that the decreased combined levels of SERT observed by some investigators may be the result of increased cleavage rather than decreased production. In addition, we confirmed the presence of two truncated N-terminal SERT proteins starting from amino acids LSVI (90) or LSVL (292) of human SERT. Finally, we demonstrate that activity of SERT correlates with the level of a cleaved N-terminal SERT isoform. Sequencing of the 35–50 kDa SERT isoform confirmed the presence of 2 potential phosphotyrosine sites at 142, and 144. Tyrosine phosphorylation of SERT at 144 regulates serotonin transport (Annamalai et al., 2012), therefore phosphorylation of 35–50 kDA SERT may have developmental implications. Further studies are required to clarify the sources as well as the biological significance of the observed heterogeneity of SERT-immunoreactive species.

The specific mechanisms guiding developmental differences in SERT levels are not well understood. Non-GA-dependent downregulation usually occurs through a combination of the effects of Protein Kinases C and G, increased intracellular Ca^2+^, NOS, and Mitogen Activated Protein Kinase (MAPK). Specific to the placenta, micro-RNAs may also play a role as miR-15a and miR-16 downregulate SERT expression in placental cells *in vitro* (Moya et al., 2013). Maternal mood may alter placental SERT gene expression (Ponder et al., 2011) in the 3rd trimester; however, it is unlikely that this is a significant driver of gestational changes. Cytokines, especially the interleukin-1 family, and TNF-alpha increase SERT gene expression and serotonin transport activity *in vitro* (Ramamoorthy S. et al., 1995; Kekuda et al., 2000; Zhu et al., 2006). The increase in pro-inflammatory cytokines at full term, when most 3rd trimester placental samples are collected, may partially explain the sharp increase in SERT that we observed (Goetzl et al., 2002; Unal et al., 2011; Boles et al., 2012; Cierny et al., 2014).

### NET

NET is primarily thought to mediate placental catecholamine clearance at the basal membrane of the syncytiotrophoblast (Ramamoorthy et al., 1993b). Little has been published regarding placental levels of NET over gestation although NET was known to be present in the placenta (Schroeter et al., 2000; Jayanthi et al., 2002; Sung et al., 2003). We present data that NET is present in purified apical membrane fractions, but does not appear to be uniformly developmentally regulated. A small increase in NET was noted in 2nd trimester samples, with uncertain significance for drug transport. Our focus on the apical brush border may have affected the overall picture of NET, as NET activity is likely to be focused in the basal membrane (St-Pierre et al., 2002). Further investigation of developmental regulation of NET in basal membrane fractions is planned. Alternatively, the lack of GA variation in NET expression may serve to stabilize fetal catechol levels. At term, NET RNA expression is inversely associated with cord blood norepinephrine levels (Bzoskie et al., 1997).

Our finding of NET in the apical membrane may have important implications for drug interactions. We have previously reported that cocaine up-regulates NET in placental tissue by increasing NET surface availability (Mannangatti et al., 2011). Therefore, our finding of NET on the apical membrane suggests that maternal cocaine use may increase maternal-fetal influx of norepinephrine, amphetamines and other xenobiotics across gestation. In contrast, we have also reported amphetamine-mediated down-regulation of placental NET is thought to occur through reduced apical membrane expression and enhanced endocytosis (Annamalalai et al., 2012). More work is needed to quantify the effects of early and late exposure to amphetamines and cocaine on placental NET.

### BCRP

BCRP is an efflux drug transporter located in all areas of the human term placenta (Allikmets et al., 1998; Kozlowska-Rup et al., 2014). It is primarily located on the apical membrane of the syncytiotrophoblasts but also in the endothelial cells lining the fetal capillaries (Ceckova et al., 2006). There have been conflicting reports published on placental BCRP expression across gestation. Mathias et al. reported that placental BCRP levels at various GAs were variable and did not appear to change with gestation (Mathias et al., 2005). Yeboah et al. investigated BCRP mRNA and protein expression in the 1st and 2^nd^ trimesters as well as several GA strata in the 3rd trimester (Yeboah et al., 2006). They did not see a significant GA pattern but they did report an increase in BCRP expression in fetal blood vessels in the 3rd trimester compared to the 1st trimester. Both of these investigations were significantly limited by the very small number of samples interrogated and their use of whole placental extracts. In contrast, in a much larger study, Meyer zu Schwabedissen et al. reported that BCRP protein and mRNA was higher in the early 3rd trimester compared to the late 3rd trimester; earlier GAs were not included (Meyer zu Schwabedissen et al., 2006). Anoshchenko reported decreasing protein levels of BCRP in placental membranes with increasing gestation (Anoshchenko et al., 2020). In the current manuscript, we report on BCRP levels in a large number of healthy samples in each trimester, increasing our statistical power and compensating for the high level of natural variation in human samples. We confirm Anoshchenko’s findings that BCRP levels fall across GA. Our findings suggest that BCRP may play a similar role to P-gp in the early protection of the fetus from xenobiotics. Of note, the common use of cannabinoids in the 1st trimester of pregnancy may inhibit BCRP, limiting protection of the developing fetus from BCRP substrates (Feinshtein et al., 2013).

### MRP3

MRP3 has been previously demonstrated in human placenta (St-Pierre et al., 2000) but almost nothing has been published about MRP3 levels across gestation. We report here that MRP3 levels are stable in the 1st and 2nd trimesters, but that there is a significant upregulation of the 160 kDa isoform in the 3rd trimester. The mechanism for this upregulation is not known but may be related to 3rd trimester increases in inflammatory mediators. TNF-alpha can modify the expression of multi drug resistance associated proteins in hepatic cells *in vivo* (Bohan et al., 2003; Chai et al., 2012). Clinically, increased 3rd trimester MRP3 may serve to partially protect the fetus from bile acids in the setting of maternal cholestasis and from opioids in labor.

### P-gp

Our findings that P-gp falls precipitously across GA confirm multiple prior reports (Gil et al., 2005; Mathias et al., 2005; Sun et al., 2006; Anoshchenko et al., 2020). Therefore, while the findings presented here are not novel, they serve to validate our methodology related to the other transporters of interest. MacFarland et al. also demonstrated differences in distribution of P-gp with predominantly trophoblast expression in the 1st trimester and macrophage expression in the 3rd trimester (MacFarland et al., 1994). We present the largest cohort to date with accurate quantification of P-gp expression across all three trimesters. Our data, along with previously presented data, adds to the increasingly strong evidence that P-gp serves an important protective role in early gestation. At this time, there is some direct corroborating evidence from specific fetal drug levels across human gestation. Nanovskaya et al. were able to demonstrate that *in-vivo* placental transfer of methadone, a P-gp substrate is lower in preterm placentas compared to term placentas suggestion increased P-gp efflux protection at earlier gestations (Nanovskaya et al., 2008). However, this protection may be offset in non-naïve placental tissue as opiates inhibit P-gp activity in inside out placental vesicles (Hemauer et al., 2009). In addition, the C3435T Polymorphism of P-gp (which results in decreased levels of P-gp) in conjunction with phthalate exposure in early gestation, increases the risk for congenital heart disease (Wang et al., 2013). This demonstrates the critical role of P-gp in early gestation in protecting the fetus against potentially teratogenic substances.

The mechanism for GA dependent regulation of P-gp is not well understood but because similar patterns are observed in both protein and P-gp transcripts, it is hypothesized that the primary regulation is at the level of transcription Progesterone has been reported to increase P-gp expression in animal models (Lee et al., 1998), however progesterone levels increase with pregnancy, while P-gp levels fall; therefore a stimulatory role of progesterone would not explain the inverse relationship observed. Similarly, estradiol (E2) increases P-gp expression in placental cell lines (Coles et al., 2009). However, since E2 also increases with gestation, this would not explain the observed GA decreases in P-gp. Mathias et al. commented on the similar pattern between human chorionic gonadotropin (hCG-β) and P-gp however they did not demonstrate a causal relationship (Mathias et al., 2005). Finally, hypoxia is associated with an increase in P-gp in 3rd trimester placental samples (Javam et al., 2014). Therefore intrauterine growth restriction, and other conditions thought to be associated with chronic placental hypoxia, may boost fetal protection against medication exposures.

### Strengths and Weaknesses

The important strength of the current manuscript is our expertise in obtaining healthy placental tissue across gestation from a large number of subjects. Our rapid collection process resulted in high quality tissues with active protein activity as demonstrated by ALP. Further, our methods, which relied on protein quantification in the membrane fraction of the placenta, focus comparisons between trimesters on the functionally important area of placental transport and reduces confounding by increased levels of non-functional connective tissue in the 3rd trimester. Use of quantitative westerns further increases the validity of comparisons. Finally, our use of tissues from women who underwent a structured drug interview is critically important given the known effects exposures to drugs and medications such as cannabanoids, SSRIs, amphetamines and others on drug transporter levels. The common use of non-characterized samples of convenience or commercially obtained samples is subject to considerable confounding given the significant rates of drug and medication use in women undergoing 1st and 2nd trimester pregnancy termination.

There are few potential weaknesses to human placenta studies. Lanoix et al. have demonstrated that well-accepted loading controls (alpha-tubulin and GAPDH) may not be the ideal placental references proteins to compare disease states in the 3rd trimester (Lanoix et al., 2012). They suggest general protein stains as a more reliable benchmark; normalizing to alpha-tubulin may slightly but systematically underestimate SERT levels. To offset this concern, we used Grb2 as a loading control, in addition to protein gel staining but systematic underestimation of SERT, if consistent, is unlikely to affect our results as all of our results are relative. Finally, our study was underpowered to effectively study polymorphisms of transport proteins or their promotors and their effect on placental protein levels.

## Conclusion

Taken as a whole observed patterns of SERT, NET, P-gp, BCRP and MRP3 serve to protect embryonal development at early trimesters and to decrease placental permeability in the 1st trimester to transporter specific substrates including commonly used medications such as anti-depressants, anti-psychotics, amphetamines, protease inhibitors, and some antibiotics. Overall our observations are consistent with a strong protective effect during organogenesis. Further, 3rd trimester estimates of fetal exposure obtained from cord blood likely significantly overestimate early fetal exposure to these medications at any fixed maternal dose. Our use of vesicles for protein quantification is an important step; our group and others have used placental vesicles to quantify substrate transport for substrates of SERT, NET and P-gp (Ushigome et al., 2003; Rahi et al., 2007; Rubinchik-Stern and Eyal 2012). ROV or IOV can also be used to study competitive inhibition and other drug-drug interactions in the setting of multiple drug exposures (Glavinas et al., 2008). Therefore, a natural next step is to investigate *in-vitro* transport of specific substrates across GA in drug naïve placentas (controls) and in placental tissue collected from women with chronic exposure. Continued research into both the mechanisms regulating expression of key placental drug transporters and the downstream functional result of altered transporter function and activity is critical in the development of thoughtful strategies to simultaneously minimize fetal drug exposure while optimizing drug therapy for pregnant women.

## Data Availability

The raw data supporting the conclusion of this article will be made available by the authors, without undue reservation.
